# Where Am I? Location Archetype Keyword Extraction from Urban Mobility Patterns

**DOI:** 10.1371/journal.pone.0063980

**Published:** 2013-05-21

**Authors:** Vassilis Kostakos, Tomi Juntunen, Jorge Goncalves, Simo Hosio, Timo Ojala

**Affiliations:** Department of Computer Science & Engineering, University of Oulu, Oulu, Finland; MIT, United States of America

## Abstract

Can online behaviour be used as a proxy for studying urban mobility? The increasing availability of digital mobility traces has provided new insights into collective human behaviour. Mobility datasets have been shown to be an accurate proxy for daily behaviour and social patterns, and behavioural data from Twitter has been used to predict real world phenomena such as cinema ticket sale volumes, stock prices, and disease outbreaks. In this paper we correlate city-scale urban traffic patterns with online search trends to uncover keywords describing the pedestrian traffic location. By analysing a 3-year mobility dataset we show that our approach, called Location Archetype Keyword Extraction (LAKE), is capable of uncovering semantically relevant keywords for describing a location. Our findings demonstrate an overarching relationship between online and offline collective behaviour, and allow for advancing analysis of community-level behaviour by using online search keywords as a practical behaviour proxy.

## Introduction

Research has shown that the study of urban mobility can offer insight into human behaviour [1]–[3]. One mechanism that makes this possible is the concept of a *location attractor* [4] in urban environments, i.e. that certain locations attract individuals exhibiting a particular behaviour deviating from routine movement. For instance, a sports stadium becomes such an attractor when it hosts sports events, causing individuals interested in sports to alter their daily routine and visit the stadium. Along the same lines researchers have also attempted to understand the relationship between mobility and behaviour by linking mobility traces corpuses to geospatial configuration [5].

However, capturing urban mobility traces is challenging and often requires infrastructure that can be expensive and complex to maintain. For this reason, researchers have increasingly studied online behaviour datasets to uncover patterns in collective human behaviour. Online datasets are convenient to capture and analyse, and previous work has already noted their potential. For example, trends in online search activity have been shown useful in providing models of real world phenomena such as influenza outbreaks, stock market activity, and consumer behavior [6]–[10]. One example is the search volume of the keyword “flu” which correlates with the outbreak of influenza as recorded in national health statistics [6], [8], [11]. These models typically rely on the careful choice of search keywords that are expected to correspond with a particular real-world phenomenon.

In this paper we set out to investigate the relationship between urban mobility and online keyword search volume. Because *both* urban mobility [1]–[5] and keyword search volume [6]–[10] correlate with collective behaviour, we hypothesise that they may be linked directly. Evidence of such a relationship would suggest that urban mobility can be studied by using online data as a proxy, provided that the appropriate keywords can be identified.

In this paper we make a number of contributions. Specifically:

- We show that urban mobility patterns can be modelled using a set of search keywords. This means that the mobility at particular locations can be modelled using the popularity of certain keywords over time.
- We show that these keywords appear to be semantically relevant to the respective locations. This suggests a strong relationship between urban mobility and online search behaviour.
- We fully describe our process (called Location Archetype Keyword Extraction, or LAKE), which relies on publicly available tools. This means that other researchers can immediately validate our approach by using LAKE with their own data.
- We demonstrate that the reason for LAKE’s effectiveness is the existence of location archetypes. This means that mobility across semantically relevant locations correlates highly, and therefore LAKE identifies keywords that are relevant to semantically similar locations.
- We also provide evidence that the correlation of mobility at any two locations is also geographically bound, specifically inversely related to their distance.

The implications of our findings are diverse. First, we provide evidence of a link between urban mobility and online search behaviour. Furthermore, our work presents a cheap way to estimate pedestrian mobility at urban locations using search keywords as a proxy. Finally, our findings show that our method can also characterise urban locations given mobility data, which is a valuable input for interactive systems operating in urban environments.

## Materials and Methods

### Data Provision

Our approach uses online search data provided by Google. According to analytics firm NetMarketShare, Google’s share of global online searches was 84.14% in November 2012, making it the biggest online search engine in terms of volume of executed searches. Thus, we argue it provides the most comprehensive search data for us.

*Google Trends* is a publicly available online tool by Google that provides insights into temporal and spatial volumes of search queries from the search engine. Its main purpose is to model over time the popularity of keywords used in search queries. Building on top of this tool, *Google Correlate* can identify web search queries whose temporal frequency best correlates with any user-specified temporal pattern [12]. Google Correlate requires a time series data in date-value pairs as an input. The format of the input data is a two-dimensional vector of form *[dd/mm/yyyy, value].* The tool then attempts to find search queries whose temporal patterns match the input data. The tool operates on a national level, such that the popularity within a particular nation is considered.

We independently collected urban mobility traces using a network of WiFi access points across our city. An access point was installed in each of the locations in our study, and we calculated the number of unique devices detected by the access point per day of the study. We normalise this data and use it as an approximation of the pedestrian flows in various locations. One pedestrian flow vector can therefore be used to describe pedestrian activity at one location for the duration of our study. Using one such vector as an input, the Google Correlate tool provides a list of the top-10 search keywords whose popularity best matches the pedestrian flow vector over time. The top search keywords are ranked in terms of the correlation’s *r* coefficient.

### Data Collection

**Figure 1:**
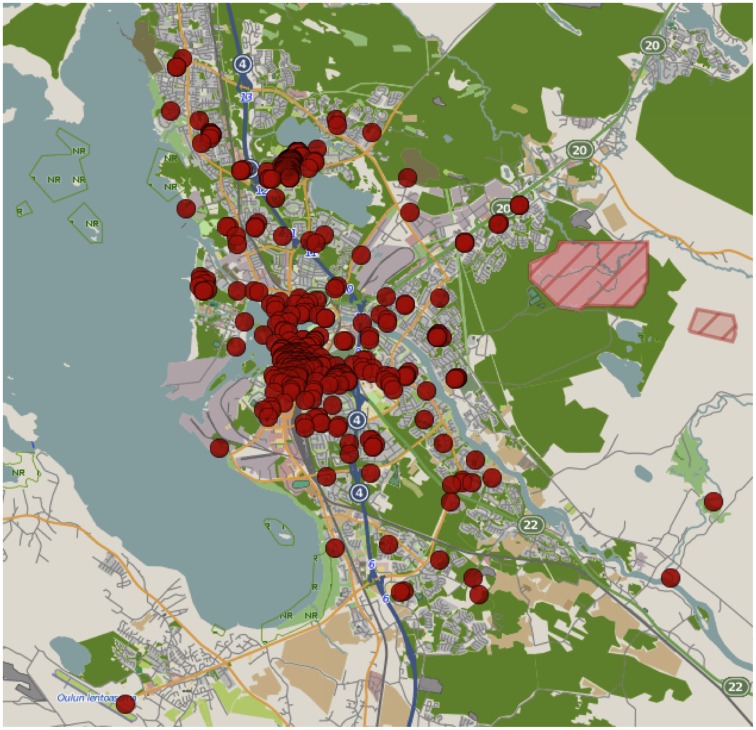
A map showing all the WiFi access point locations. The map covers an area approximately 20km×20km.

To investigate the relationship between urban mobility and online search activity we collected a longitudinal dataset of city-scale mobility, and used the Google Correlate tool to match time-series mobility data with search keywords. We collected mobility traces using a municipal WiFi network between January 2009 and May 2012. The network consists of 1300 access points covering a large portion of the area of the city of Oulu, Finland (shown in Figure 1). The network is free, open, and available in a number of public locations including schools, libraries, health clinics, and public squares.

**Figure 2:**
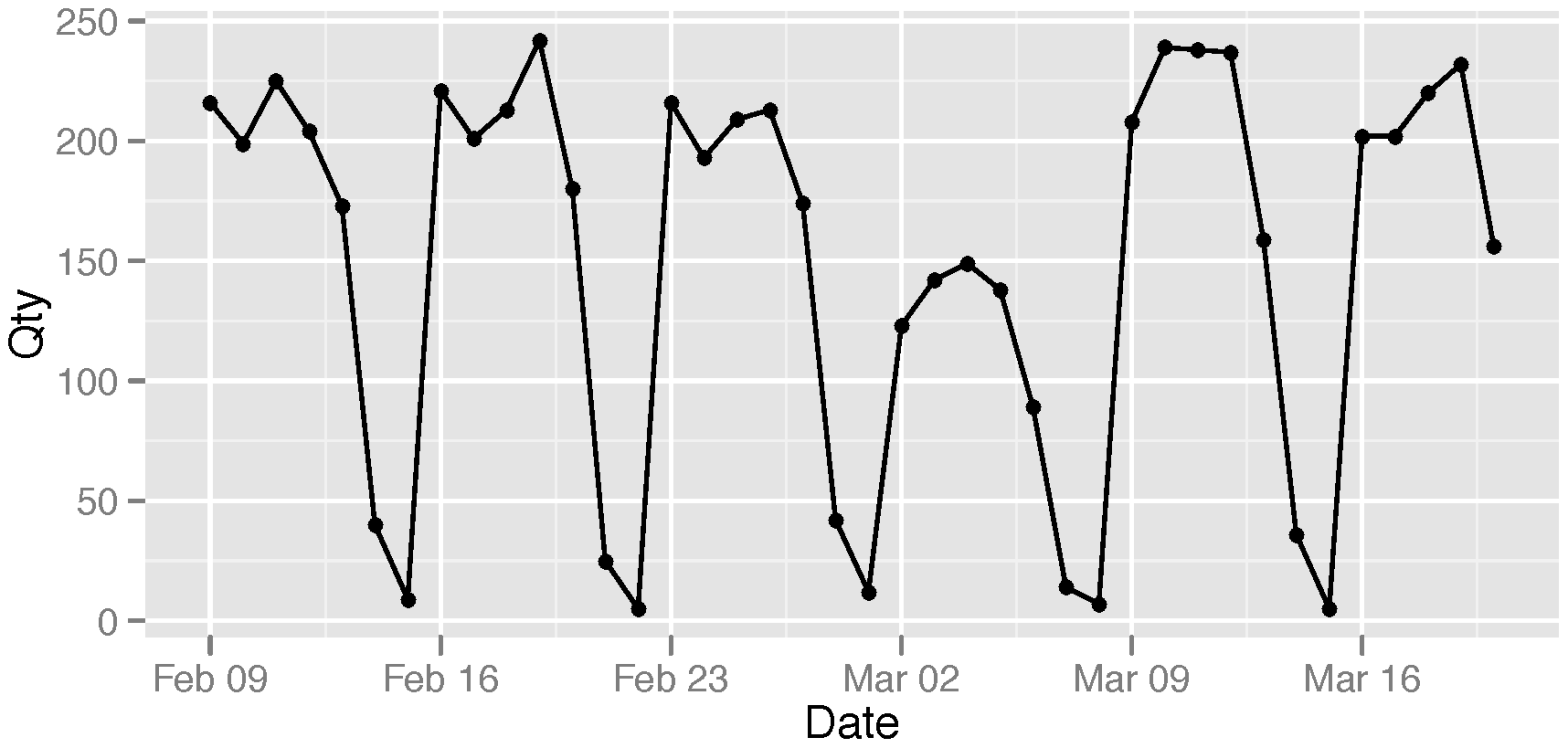
Total number of unique devices detected at one location. The data demonstrates how over a period of 4 weeks in 2009 the number of devices fluctuates on a daily pattern. The first data point in this plot is a Monday. The data also shows that weekends have distinctly less activity.

We also calculated the daily number of visitors at a variety of locations by considering the number of unique devices that used the WiFi network at a particular location. For example, Figure 2 shows the number of unique devices detected in our University over a period of 4 weeks in 2009. This analysis is possible because each device uses a unique identifier, and each of the 1300 access points is uniquely identifiable by our software. Each device that accesses the network is assigned a unique identifier (ID). If a device connects to the network for the first time, the device ID will be determined based on the MAC address of the device using a one-way hash function. When a device connects to the network, our software records the device ID, the date and time, and ID of the WiFi access point that can be used to infer the location. The ID of a device or access point does not change over time. Thus, we were able to determine how many unique devices have accessed the network at specified location on each day for a given time period. A limitation of this approach is that if a user has multiple WiFi devices, our analysis will infer that multiple users have accessed the network.

Some locations have multiple overlapping access points installed to cope with high network demand. In these cases, our analysis treats those access points as an aggregate “virtual” access point. This ensures that a device is only recorded once in that area or building, even though it may actually associate with multiple access points. We manually inspected the locations in our analysis and we defined virtual points in this manner. For example, all access points across the 3 floors of the library were aggregated into a single virtual access point, and similarly the lobbies in our university were all aggregated into a single virtual access point. The same is true for the access points high schools, where we coded the data so that each high school is a single virtual point. Overall, we defined 12 virtual access points in our analysis representing 84 real access points.

### Visual Interpretation of the Data

**Figure 3:**
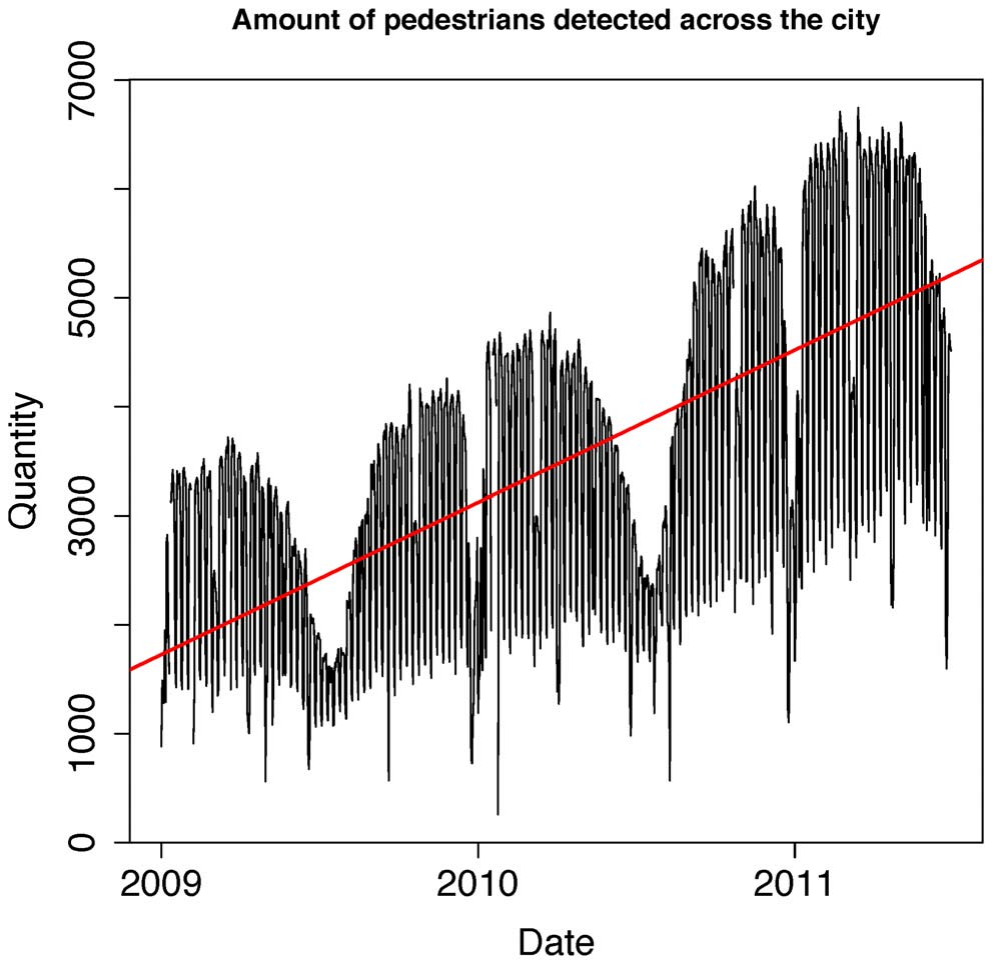
Total number of unique devices detected in the city during the study period. The data demonstrates how over a period of three years the volume of devices doubles, much unlike the population of our city that has grown at more modest rates.

We are able to generate time-series data describing the number of visitors across the city as well as at a particular location, for example at our University. The total number of devices detected across the city during the whole study is shown in Figure 3, and clearly demonstrates an upward trend underlying the varying seasonal patterns. We hypothesize that this upward trend is due to the increasing penetration of WiFi enabled lightweight devices such as smartphones and tablets during the study period [13].

**Figure 4:**
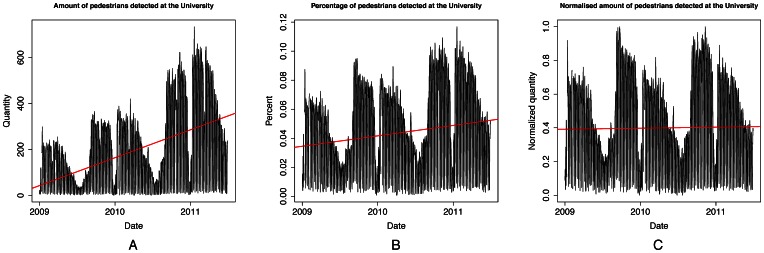
Normalisation of pedestrian flows time series. Figure 4a shows the number of WiFi devices detected during the study period in the lobby areas of our university. The data demonstrates strong seasonal patterns, for example showing that summer time is relatively quiet at the University. At the same time, however, this time series shows an upward trend, reflecting the increasing number of WiFi devices using the network across the city over time. To account for this increasing trend while maintaining the seasonal patterns, we normalise the data using a two-step approach. First we calculate the percentage of all devices seen on any day that visit a particular location on that day. Results of this normalization step are shown in Figure 4b. Subsequently, we apply an annual minimum-maximum normalisation filter to derive the time series in Figure 4c. The effectiveness of our normalization method is verified using linear regression (in red), which shows that compared to the original data (*x = 0.3324, r^2^ = 0.2192, p<2.2e-16*) the normalization in Figure 4b (*x = 1.964e-05, r^2^ = 0.04205, p = 3.318e-12*) and subsequently Figure 4c (*x = 1.724e-06, r^2^ = *–*0.000879, p = 0.9438*) retain mostly seasonal patterns in variation.

The time-series data for a particular location, our University, is shown in Figure 4 along with a normalisation process aimed at retaining the seasonal patterns but discarding the overarching upward trend in the number of WiFi devices detected. This upward trend affects the results obtained in our analysis, because in addition to the seasonal patterns it also takes into account the upward trend of the data. As a result, search queries that have become increasingly popular since 2009 are ranked higher when we conduct our analysis using just the raw pedestrian flow data. Because our analysis is more focused on the seasonal patterns of the data, we perform the normalization process described next.

### Data Normalization

We illustrate our normalisation procedure using the data collected from the main lobby areas of our University. Figure 4 visualizes the effects of our normalization method. The pedestrian flow data shown in Figure 4a was collected in our University, and shows that the beginning of the semester in autumn is relatively busy when freshmen start their studies, while the amount of students decreases gradually during the year. A sharp drop in the amount of pedestrians can also be seen during the Christmas holidays.

To filter out the upward trend component and maintain the seasonal patterns, we denote the university time-series data as a vector *K*, where a value *K_i_* is the number of devices detected on *i^th^* day of our study in that location. The vector *T* is a vector where value *T_i_* is the total amount of devices detected on *i^th^* day in the whole city.

Therefore, the percentage of devices across the whole community that were detected in the university is
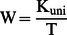
where *W* is a vector containing the relational frequency of the detected devices for each day of the study. The result of this normalization is shown in Figure 4b but still entails an upward trend. Given the strong annual pattern across all our data, we apply minimum-maximum normalization on each year separately to highlight the seasonal variations. The resulting normalized vector can be denoted as



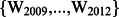
where *W_year_* is a minimum-maximum normalized annual subset from the vector *W* and is denoted as



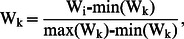


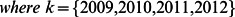


This normalization step further reduces the upward trend component, and the results are shown in Figure 4c.

To validate our normalization method we rely on linear regression and inspect the slope *x_i_, r^2^* and *p-value* of the regression lines in Figures 4a, 4b and 4c. This approach was used to validate whether the upward trend component, estimated with a linear regression line, can be progressively eliminated. Our aim is to derive a “stable” oscillating function with a linear regression line whose slope is near zero and has low *r^2^* value. The results of this validation process are presented in the results section. Note that our intention is to verify that a linear regression has a *poor* fit, thereby suggesting that the linear trend in the data has been largely eliminated.

### Keyword Extraction

Using our normalisation approach we derive one time series per location of interest in the city, and use each one to identify search queries with similar temporal patterns on Google Correlate. Effectively, we uploaded pairs of values of the format <date, value>, using each day as a data point. Google correlate aggregates this data into weekly values. Our analysis results in the top 10 online search keywords used in Finland whose search volume time series matches the pattern of pedestrian flows (using Pearson’s correlation) at each location respectively.

Our results show that, when appropriate keywords are used, the pedestrian flows at a location can be approximated with a degree of accuracy of 0.7–0.9. However, our results did have an unexpected element. Surprisingly, the results were keywords that appeared to be semantically relevant to the locations. For example, given the time series from the University data (Figure 4c) some of the keywords we obtained are “research”, “scholar” and “lecture” (Table 1). These terms were derived by feeding the normalised data for each location to the Google Correlate tool and deriving the top-10 search terms whose popularity matches the pedestrian flow volume. We refer to this whole process as Location Archetype Keyword Extraction (LAKE).

**Table 1:** Top 10 search engine queries for various locations.

University	r-value	Ice hockey hall	r-value
Helka (Helsinki university library)	0.914	sjl (Finnish ice hockey union)	0.726
google scholar	0.895	finnhockey	0.724
scholar	0.894	keilahalli (bowling hall)	0.722
tutkimus (research)	0.893	kiekko kaleva (hockey in local newspaper)	0.716
learning	0.887	sm-liiga nelonen (tv-channel with hockey)	0.715
optima oulu (student environment)	0.878	nelonen sm (same as above)	0.713
funktio (function)	0.876	jääkiekkoliitto (ice hockey union)	0.711
luento (lecture)	0.874	nelonen sm liiga (tv-channel, ice hockey)	0.707
development	0.872	lihapata (meat stew)	0.699
nelli (university e-library portal)	0.871	finhockey	0.699
**Library**	**r-value**	**High school**	**r-value**
hietsun kirppis (flea market)	0.889	wilma kempele (student environment)	0.795
pistiäinen (stinging bee)	0.870	wilma kiiminki (student environment)	0.791
viinitila (wine farm)	0.864	helmi (student management system)	0.781
reitti (path)	0.863	wilma oulu (student environment)	0.771
lättähattu (50′s dressing style/old train)	0.862	edu (news and info on education)	0.763
hietaniemen kirpputori (flea market)	0.860	wilma kuusamo (student environment)	0.758
hietalahden kirpputori (flea market)	0.859	pedanet (support for online learning)	0.747
museorautatie (museum railroad)	0.857	wilma raahe (student environment)	0.744
korppoo (island in Turku archipelago)	0.854	wilma kemi (student environment)	0.736
tammisaari (town)	0.853	varoitusmerkit (warning signs)	0.726
**Camping**	**r-value**		
festivaali (festival)	0.804		
naantali majoitus (housing)	0.793		
kalajoki camping	0.788		
hanko majoitus (housing)	0.761		
vesipuisto serena (water amusement park)	0.760		
rauhalahti camping	0.759		
rengastie kartta (map)	0.758		
Högsåra (island in Hittinen archipelago)	0.756		
kuhan uistelu (zander fishing)	0.740		

The r-values reported are calculated byGoogle Correlate, and here we report the top-10 results for each location. In brackets are English translations where necessary.

To verify that the results of LAKE were indeed semantically relevant to the respective locations, we issued a questionnaire to 29 residents of our city (22 male, 7 female) with an average age of 30.5 (*σ = 9.5*). Each respondent was given the following:

- a set of location names (High school, University, Library, Camping, Ice Hockey Hall)
- a set of 16 keywords
- instructions to rate on a 5-point likert scale the semantic similarity of each keyword to the respective location.

We constructed the set of words for each location by aggregating and mixing.

- the top 10 keywords for that particular location derived using our analysis
- 3 words selected at random
- 3 keywords from a different location.

Thus, each of the 15 respondents was given 5 locations and 16 words per location. The order of words for each location was randomized. Respondents rated each word on a 5-point Likert scale to indicate the semantic relevance of that word to the respective location. No information was given to respondents about how the words were derived, the respondents were recruited via email, and all were natives and residents of our city. The respondents were instructed to consider the broader context of the locations, for instance the search term “marketing” refers to “marketing” as university studies (economics) or “product marketing”. The respondents were also encouraged to use online search if they were unfamiliar with any of the queries. Locations were identified only as their categorical names. No more details about the locations or their whereabouts were given. Using this method, we hypothesise that the top 10 keywords included within the scrambled set of 16 words will receive higher ratings than the set of random keywords, suggesting that LAKE produces semantically relevant results. Furthermore, we hypothesize that the top 10 keywords will receive higher rating that the 3 keywords from a different location, suggesting that LAKE distinguishes between locations. As we describe in the results section, both our hypotheses were confirmed by the independent respondents.

### Interpretation of LAKE Analysis

The mechanism that enables LAKE to produce semantically relevant keywords – without direct human intervention – is not immediately evident. Besides the lack of direct human input, we were puzzled by the fact that pedestrian flows are collected at a *particular location* in our city, while the Google Correlate tool operates on a *national level*. Given our findings, we hypothesise that there must exist “location archetypes”, instances of which are the particular locations we observed with our WiFi network. For example, we hypothesise that our university must be just one of many instances of the “University archetype”, and therefore nation-wide online search queries must correlate with the pedestrian flows at all instances of this location archetype. For this reason we refer to the process we have described so far as Location Archetype Keyword Extraction (LAKE), because we hypothesize that it allows us to extract keywords referring to the archetype of locations we study.

**Figure 5:**
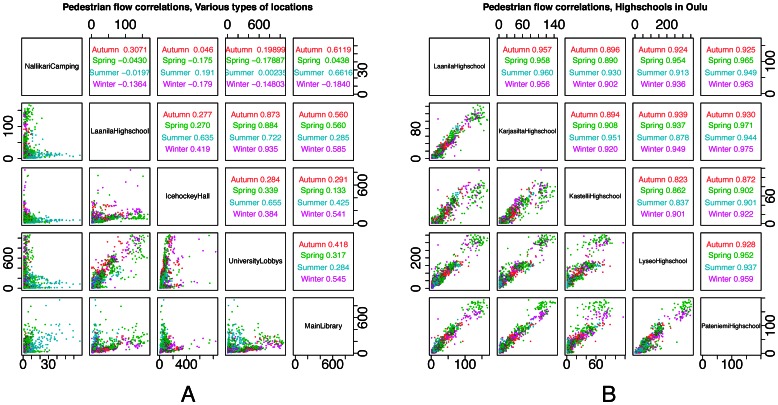
Scatterplot matrices showing pedestrian flow. Here we can see pedestrian flows for (a) various types of locations and (b) high schools. In this figure each scatterplot is for a pair of locations. Each scatterplot data point is for a particular day of our study, and indicates the correlation of pedestrian flows for the two locations on that particular date. Data points are color-coded by season to account for seasonal variations. Reading the scatterplots: to locate the scatterplot for a pair of locations, the row-column intersection cell need to be inspected below the diagonal. Similarly, the correlation values are at the row-column intersection cell above the diagonal.

To investigate the existence of location archetypes, we analyse longitudinal pedestrian flows collected from various locations that we intuitively believe must belong to the same archetype: five high schools in our city. The correlation of daily visitors at a number of unrelated locations is shown in Figure 5a and contrasted with the data for five distinct high schools in Figure 5b. The results demonstrate that pedestrian flows at all the high schools correlate strongly with each other (*r>0.8*), and thus are likely instances of a “High School archetype”. Subsequently, the pedestrian flow data from all high schools produce semantically relevant keywords using our LAKE analysis.

This analysis relies on daily flows of pedestrians at various locations. In our analysis we also considered using hourly-intervals to compare locations, but the results were unreliable. When considering hourly intervals, the Circadian rhythm becomes very prominent. In other words, the “nominal” daily patterns take over, with high activity during work hours and low activity during nighttime and weekends. For this reason the analysis results in high correlation patterns between most locations, and effectively reduces the ability of our technique to differentiate between locations.

### Distance Effect on Correlation

An alternative explanation for the results in Figure 5 on the correlation of pedestrian flows may be distance: the high correlation in urban mobility between two locations may be due to their physical proximity. This is a spatial phenomenon that has previously been hypothesised [4]. We investigate whether the strong correlations we identified may be due to spatial proximity in addition to the existence of location archetypes. Because the LAKE analysis operates on pedestrian flows, we expect that locations close to each other are likely to strongly correlate with each other regardless of their semantic relationship. Effectively, we assume that a group of people moving across the city is likely to register at multiple locations that may not be semantically relevant to each other but be spatially proximal to each other.

To investigate this relationship, we constructed two matrices: a distance matrix *M_D_* shown in Table 2 and a correlation matrix *M_C_*, which can be seen in Table 3. The distance matrix has all the locations of the study denoted with *L_i_* as rows and columns, and their respective distances *D_ij_* from each other. The correlation matrix is structured in a similar manner where correlation is denoted with *C_ij_*. The diagonals of both matrices are left empty.

**Table 2:** Distance matrix for all locations.

Location	L_1_	L_2_	…	L_j_
**L_1_**	–	D_12_	…	D_1j_
**L_2_**	D_21_	–	…	D_2j_
**…**	…	…	–	…
**L_i_**	D_i1_	D_i2_	…	–

Each cell *D_ij_* denotes the distance in meters between locations L_i_ and L_j_.

**Table 3:** Pedestrian flow correlation Matrix for all locations.

Location	L_1_	L_2_	…	L_j_
**L_1_**	–	C_12_	…	C_1j_
**L_2_**	C_21_	–	…	C_2j_
**…**	…	…	–	…
**L_m_**	C_i1_	C_i2_	…	–

Each cell *C_ij_* denotes the Pearson’s correlation in daily pedestrian flows between locations L_i_ and L_j_.

**Figure 6:**
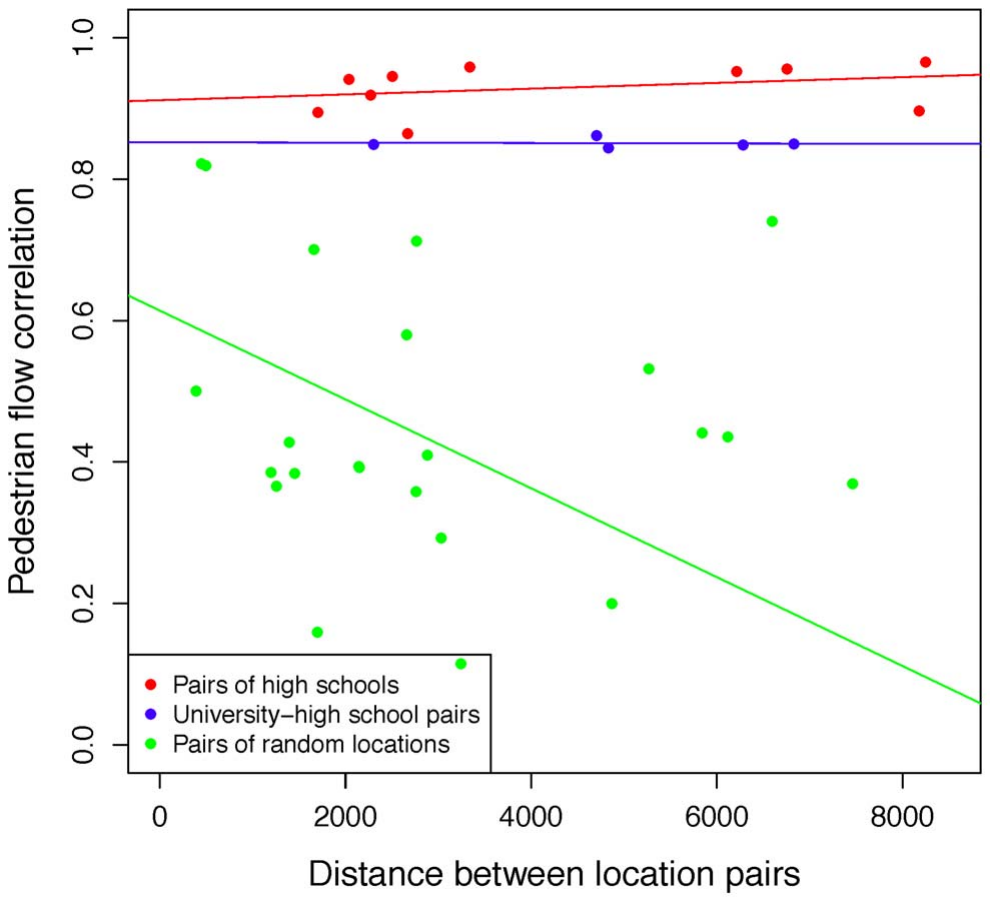
Distance affects pedestrian flow correlations. Correlation in pedestrian flows is affected by distance (in meters) between two locations. Orange dots are pairs of high schools, and blue dots are pairs consisting of the university and high schools. Green dots are pairs of semantically irrelevant locations. Regression lines are included with the colour of the respective category. We identify two trends in this data. With the green colour we show location pairs that are not semantically relevant, which demonstrate an inverse effect between distance and correlation of pedestrian volumes (*x_random_ = *–*6.285e-05, r^2^_random_ = 0.11, p_random_ = 0.007*). In orange we show location pairs that are semantically relevant (pairs of high schools) and in blue we show location pairs that are highly related to each other (pairs consisting of the university and one high school). We find that for both sets of pairs distance has no significant effect on the pair’s correlation of pedestrian flows (*x_university_ = *–*2.647e-07, r^2^_university_ = *–*0.327, p_university_ = 0.910; x_highschools_ = 4.075e-06, r^2^_highschools_ = 0.051, p_highschools_ = 0.172*).

We then use these two matrices to construct the scatterplot in Figure 6, where each point in the scatterplot can be denoted with coordinates (*D_ij_, C_ij_*) from the two matrices. In this figure each data point is for a pair of locations. For each pair we use the Pearson’s r correlation in pedestrian flows (Table 4) and the physical distance between those two locations in meters (Table 3). We then plot these two values along the *y* and *x* axes of the figure respectively. In addition we use colour coding (green-blue-red) to indicate an increasing expected semantic similarity that we expect for those pairs. Our results confirm that distance is inversely related to the correlation between locations, but only for locations that are *not* semantically relevant. In the latter case, distance has little impact on the correlation.

**Table 4:** Summary of the linear regression analysis.

Figure	r^2^	x_i_ (slope)	p-value
3	0.31250	3.3830	<2.2e-16
4a	0.21920	0.3324	<2.2e-16
4b	0.04205	1.964e-05	3.318e-12
4c	−0.00879	1.724e-06	0.9438

Values of the normalization procedure from each dataset in Figures 3 and 4.

## Results

In Figure 3 we verify that the total number of unique WiFi devices detected across the city has indeed an upward trend highlighted in red (*x_a_ = 3.383, r^2^_a_ = 0.3125, p_a_<2.2e-16*), and we also verify that this trend is present in the University raw data in Figure 4a (*x_b_ = 0.3324, r^2^_b_ = 0.2192, p_b_<2.2e-16*). Our first normalisation step (Figure 4b) partially filters this trend by relying on the percentage of devices detected in the University area for each day of the study (*x_c_ = 1.964e-05, r^2^_c_ = 0.04205, p_c_ = 3.318e-12*). Our final normalisation step (Figure 4c) utilizes the annual minimum-maximum normalization and adequately filters the upward trend by resulting in a non-significant linear fit (*x_d_ = 1.724e-06, r^2^_d_ = *–*0.000879, p_d_ = 0.9438)*. We summarize the regression coefficients for each dataset in Table 4.

This normalization procedure shows that by calculating the total percentage of the devices detected in the university in relation to the whole city network, and by then performing annual minimum-maximum normalization we can filter the trend component in the data (*x_b_>x_c_>x_d_* and *r^2^_b_>r^2^_c_>r^2^_d_*). By filtering the trend component we highlight the underlying seasonal patterns of pedestrian flows in the original pedestrian flow data and thus are able to identify search engine queries and keywords that fluctuate with seasons, rather than queries that are trending over time.

The LAKE analysis was also applied to five locations of semantically different archetypes. The results of the human assessment of the search queries can be seen in Table 5. The keywords obtained through our analysis were rated consistently more relevant one a 5-point Likert scale (*α = 0.982, R_avg_ = 3.407*) than random keywords (*α = 0.948, R_avg_ = 1.620*) or keywords from different locations (*α = 0.977, R_avg_ = 2.124*). Our analysis found semantically relevant keywords for a number of locations such as high schools, a camping site, the university, and an ice hockey arena.

**Table 5:** Results of the questionnaire on LAKE analysis.

Location	Wordset (words)	Cronbach’s α	Average relevance (1–5)
Highschool	LAKE analysis (10)	0.966	3.662
Highschool	Random (3)	0.971	2.080
Highschool	LAKE from other locations (3)	0.995	2.816
Highschool	All words (16)	0.979	3.207
University	LAKE analysis (10)	0.890	4.479
University	Random (3)	0.862	1.391
University	LAKE from other locations (3)	0.975	2.678
University	All words (16)	0.986	3.563
Library	LAKE analysis (10)	0.768	1.479
Library	Random (3)	0.528	1.437
Library	LAKE from other locations (3)	0.402	2.609
Library	All words (16)	0.912	1.683
Camping	LAKE analysis (10)	0.966	3.610
Camping	Random (3)	0.969	1.701
Camping	LAKE from other locations (3)	–0.230	1.241
Camping	All words (16)	0.984	2.808
Ice hockey hall	LAKE analysis (10)	0.989	3.803
Ice hockey hall	Random (3)	0.955	1.494
Ice hockey hall	LAKE from other locations (3)	0.561	1.275
Ice hockey hall	All words (16)	0.993	2.897
**All**	LAKE analysis (10*5)	**0.982**	**3.407**
**All**	Random (3*5)	**0.948**	**1.620**
**All**	LAKE from other locations (3*5)	**0.977**	**2.124**
**Total**	All words (16*5)	**0.985**	**2.831**

Here we show for each location three sets of keywords and their respective results. Each row is an individual test case. The interrater agreement (Cronbach’s alpha) across all results was *α = 0.976* suggesting a strong agreement between the raters and the relevance or non-relevance of keywords to all the locations. For most of the cases, respondents agree that the words obtained using LAKE are more relevant to a location than words from random location wordsets or totally random words.

Figure 5 demonstrates the effect of the physical distance between pairs of locations on their correlation of pedestrian volumes. In green we show location pairs that are not semantically relevant, which as a set demonstrate an inverse effect between distance and correlation of pedestrian volumes. In blue we show location pairs that are semantically related to each other (pairs consisting of the university and one high school). Finally, in red we show location pairs that belong to the same location archetype (pairs of high schools).

We find that for location pairs that are not semantically relevant there exists an inverse effect between distance and correlation of pedestrian volumes. This is not the case for pairs of locations belonging to the same location archetype, or being semantically relevant. The results show that for the green set distance has an inverse effect on pedestrian flows correlation volumes (*x_random_ = *–*6.285e-05, r^2^_random_ = 0.11, p_random_ = 0.007*). The effect of distance is minimal in the blue and red groups (*x_university_ = 2.647e-07, r^2^_university_ = -0.327, p_university_ = 0.910; x_highschools_ = 4.075e-06, r^2^_highschools_ = 0.051, p_highschools_ = 0.172*).

**Figure 7:**
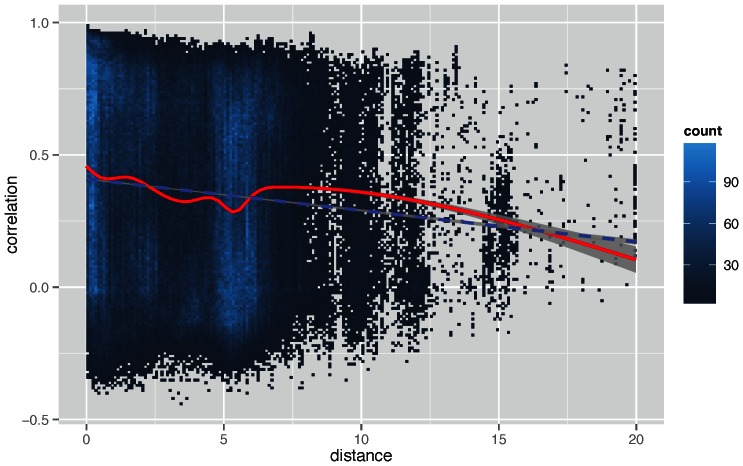
Distance affects pedestrian flow correlations in a city-scale. Correlation in pedestrian flows as affected by distance (in meters) between two locations. Approximately 300000 points contained in this figure, and contain all locations in our dataset. Blue dashed line indicates the regression between the two variables, while the red solid line shows the LOWESS smoother that uses locally-weighted polynomial regression. Both contain a confidence interval at p = 0.99. The results confirm a negative relationship between physical distance and pedestrian flow correlation amongst pairwise locations. The scatterplot contains some clustered sets of points at distance = 10, 12 and 15 kilometres, highlighting spatial clusters of WiFi access points and the polycentric nature of the region.

Finally, we constructed a scatterplot for more than 30000 random pairwise combinations of locations from our dataset. Figure 7 shows the results, which confirm an inverse underlying relationship between physical distance and pedestrian flow correlation between pairs of locations (*x_random_ = *–*1.188e-02, r^2^_random_ = 0.01012, p_random_<2.2e-16*). The scatterplot contains some vertically clustered set of points at distance = 10, 12 and 15 kilometres, highlighting spatial clusters of WiFi access points and the polycentric nature of the region.

## Discussion and Conclusion

Our results demonstrate two important findings. First, we demonstrate that LAKE can be used to identify keywords that strongly correlate with urban mobility at particular locations. This means that subsequently monitoring the popularity of these keywords can indeed offer insights into the pedestrian flows of that particular location. An important benefit of this approach is that it can be cheap and convenient to collect data on keyword popularity on large scale, thus making this analysis more accessible.

A second, unexpected, finding of our work is that the keywords produced by LAKE are semantically relevant to the respective locations. Verification with human raters suggests that indeed the keywords LAKE produced are semantically relevant to the locations. In some cases this semantic relevance is contextually derived, so we would expect only a resident of our city to be able to identify this relationship.

Furthermore, our analysis of the distance between locations and their mutual correlation shows that locations of the same archetype correlate strongly with each other regardless of the distance between them, while random locations show declining correlation as their distance increases. These findings suggest that pedestrian correlations are not only affected by seasonal variations, but show also causal relationships in a local scale.

We note that do not argue that our technique can replace existing ways of studying urban mobility. In fact, our technique relies on the existence of detailed mobility data – if this data exists in the first place, it can be used directly to conduct robust mobility analyses. What our technique complements existing urban mobility tools in two ways. First it allows us to identify keywords that can subsequently be monitored more easily than collecting urban mobility data. Second, it provides a way to derive “qualitative” data about the location in question, by generating keywords that are semantically relevant to this location. In some ways, these keywords can be understood as the broader context of the environment in which the data was collected.

### How can Location-bound Data Correlate with National-level Data?

It is not clear why location-bound pedestrian flows correlate with search engine query data, which is collected at a national level. The collected location-bound pedestrian flows represent a non-negligible part of the population of our country and the city. Even if other potential queries are correlated and compared with our data, only the relevant search queries show significant correlations (*r >0.8*).

Therefore, we find these results meaningful and believe that this phenomenon indeed demonstrates a latent relationship between online and offline community behaviour in terms of spatial and temporal activity. Specifically, we believe that the locations act as population attractors, in which the visitors’ movement frequencies and patterns create a unique “fingerprint” for each location, which becomes the main factor in the correlation process. These unique, archetype-specific pedestrian flow characteristics can then be identified using LAKE.

The findings of this paper would clearly be stronger if a larger number of location archetypes were investigated. Unfortunately there are no more location archetypes included in our data that can be reliably analyzed. This is because our data is captured from a public WiFi network that is only deployed in public spaces and governmental buildings. Many of these buildings house a mixture of facilities or services, and therefore they data we collect at those locations are not “clean”. The archetypes we have presented in this paper are all locations that have a rather well-defined purpose, and therefore our results can be reliably verified.

### Application of the Keywords’ Semantic Relevance

We have described a methodology to “convert” pedestrian flow data [14] into phrases that are semantically relevant to particular areas and contexts and also that can be used as a proxy for estimating the volume of that data. This approach can have a substantial impact on how we design and implement contextual and situated computer systems. These keywords can be used to identify relevant media, services, data, and advertisements in urban computing systems.

One straightforward mechanism to implement this is to actually use web search tools to identify relevant media. For instance, using Google’s “image search” with the queries our technique identifies, we believe that we are able to retrieve location images that are semantically relevant to the locations in our study. Similarly, our approach could be used for advertising purposes. For example Google’s advertising tools can be used to retrieve advertisements relevant to the keywords identified in our analysis. We believe that these example cases would provide substantial impact on urban pervasive computing and advertising, and therefore they need further studying and assessments by human participants.

Furthermore, our findings also point to a future direction in applying our technique. Using LAKE we can initially assign keywords to each location. Subsequently, we can conduct pairwise comparisons between locations to identify their degree of correlation, as shown in Figures 6 & 7. This degree of correlation can be used as a “weight” parameter to extend each location’s set of keywords by incorporating the keywords from the other location, coupled with a weight. Hence, for each location we can derive an extended set of associated keywords, some of which would be weighted. What this approach offers us is a way to extend LAKE by taking advantage of our findings in Figure 7: nearby locations are more likely to correlate with each other. Therefore, the extended set of keywords for a particular location could include keywords that are relevant to nearby locations as well. This is an approach that we intend to evaluate in the future.

### Limitations

Our data collection system utilizes hardware MAC addresses to identify unique devices in the network. If a *single* pedestrian is carrying or using *multiple WiFi devices* to access the network, he will be detected as if *multiple* pedestrians in the vicinity of the access point. Similarly, our method does not take into account if the detected device is stationary. For instance, residents living in the vicinity of an AP will be detected and registered as pedestrians every day, even if the AP was accessed from a residence and is stationary for the whole duration of the study. In future work these limitations could be assessed by detecting the direction from which the WiFi connection requests came to the AP and the signal strength of the connection.

Furthermore, our analysis found that adopting a different normalisation algorithm leads to some changes in the extracted keywords, particular beyond the top-20. This is not surprising since a different normalisation process results in changes to the fluctuations in data. For this reason we have followed a normalisation process described in the section “Data normalization” that does not rely on regression or opaque algorithms, but rather is based on a simple and explicit process that is justified by the nature of the data. Particularly, our normalization process looks at percentages of the whole population that visit a particular location, and also applies an annual min-max normalization step to account for incremental changes to the number of wireless devices in a city over time.

### Conclusion

By utilizing the LAKE analysis presented in this study, we have shown a way to algorithmically identify online search queries that are semantically relevant to the location where pedestrian data is collected. In addition, we have shown that locations that are close to each other are likely to have correlating pedestrian flows. As the distance between a pair of locations increases, the correlation between the pedestrian flows remains high if the locations are semantically similar. Otherwise, distance has an inverse effect on pedestrian flow correlation.

Our work enables a new approach to investigating population mobility patterns between online and offline worlds by exploiting automated data collection methods and datasets. Our findings provide insight for modelling and understanding human behaviour as reflected by urban mobility, and our technique provides a mechanism for identifying appropriate search keywords to model arbitrary real-world collective behaviour. Finally, we expect that the LAKE analysis will enable researchers to reconsider the relationship between online and physical patterns, and further contribute to the analysis of community-level behaviour.
